# ECG Changes Post-pericardiocentesis for Cardiac Tamponade Secondary to Non-small Cell Carcinoma of the Lung

**DOI:** 10.7759/cureus.54677

**Published:** 2024-02-22

**Authors:** Kyle Aldridge, Kevin E Guzman, Russell W Barry, Margaret A Franklin Christian, Felipe Ruiz, Ilya Fonarov, Damian Casadesus

**Affiliations:** 1 Medicine, St. George's University, True Blue, GRD; 2 Internal Medicine, Jackson Memorial Hospital, Miami, USA; 3 Medicine, American University of the Caribbean, Cupecoy, SXM; 4 Pathology and Laboratory Medicine, University of Miami Miller School of Medicine, Jackson Memorial Hospital, Miami, USA

**Keywords:** non-small cell carcinoma, pericardiocentesis, ecg, electrical alternans, cardiac tamponade

## Abstract

Electrical alternans on electrocardiograph (ECG) is an uncommon but nearly pathognomonic sign of cardiac tamponade. Here, we present a male quadragenarian who came to the emergency department complaining of low back and right upper abdominal pain. Work-up revealed a large pericardial effusion associated with electrical alternans on ECG and clinical findings of cardiac tamponade. Pericardiocentesis drained approximately 1 liter of hemorrhagic fluid with resolution of cardiac tamponade and normalization of the ECG. Further evaluation with right hilar lymph node biopsy confirmed a diagnosis of poorly differentiated non-small cell adenocarcinoma of the lung.

## Introduction

Cardiac tamponade is a life-threatening condition characterized by the abnormal accumulation of fluid in the pericardial space. It is common for patients to present in distress and they often complain of chest pain and dyspnea. Physical exam findings reflect the decreased filling capacity of the heart chambers, leading to tachycardia, hypotension, jugular venous distension, and pulsus paradoxus [1]. Findings on electrocardiograph (ECG) may include electrical alternans, low QRS voltage, P wave, and T wave changes [2]. Here, we showcase the resolution of electrical alternans on ECG after pericardial effusion drainage.

## Case presentation

A male in his 40s with no significant past medical history presented to the emergency department complaining of lower back and right upper abdominal pain. He reported nausea and vomiting for four days preceding the visit, along with a cough. He denied any fever, weight loss, chest pain, or shortness of breath. He did not have any history of tobacco use but engages in social alcohol consumption and smokes marijuana weekly. While in the emergency room, he was afebrile and tachycardic at 121 beats per minute and had a blood pressure of 96/64 mmHg. A cardiopulmonary exam revealed distant heart sounds. The patient had 2+ pedal edema. Abdominal exam elicited tenderness in the right upper quadrant without rebound or guarding and a negative Murphy’s sign.

Pertinent laboratory data can be found in Table 1. Interferon-gamma release assay (IGRA) QuantiFERON Gold Plus I blood test was negative. Testing for COVID-19, influenza (A and B), and respiratory syncytial virus (RSV) were negative. 

**Table 1:** Pertinent laboratory findings BUN: blood urea nitrogen, CRP: C-reactive protein, BNP:  B-type natriuretic peptide, AST: aspartate aminotransferase, ALT: alanine transaminase, ALP: alkaline phosphatase, LDH: lactic dehydrogenase, CPK: creatine phosphokinase, LDH: lactic dehydrogenase, INR: international normalized ratio

Investigation	Patient values	Reference values
BUN	23 mg/dL	6-24 mg/dL
Creatinine	1.9 mg/dL	0.7-1.3 mg/dL
CRP	5.6 mg/dL	<0.3 mg/dL
Hemoglobin	10.8 g/dL	13.8-17.2 g/dL
Hematocrit	33.7%	41-50%
Platelets	204 x 10^9^/L	150-450 x 10^9^/L
Leukocytes	10.3 x 10^9^/L	4.5-11 × 10^9^/L
Troponin	<0.012 ng/mL	<0.04 ng/mL
BNP	113 pg/mL	<100 pg/mL
AST	4608 U/L	8-33 U/L
ALT	2134 U/L	7-56 U/L
ALP	105 U/L	44-147 U/L
LDH	5,299 U/L	105-233 U/L
Total protein	5.2 g/dL	6.0-8.3 g/dL
Albumin	2.6 g/dL	3.4-5.4 g/dL
CPK	479 mcg/L	10-120 mcg/L
LDH	5,299 U/L	140-280 U/L
INR	1.81	<1.1

**Figure 1:**
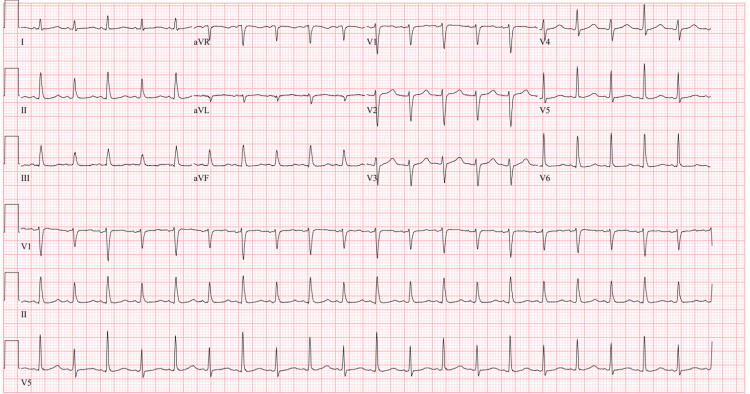
Electrical alternans on initial ECG

**Figure 2:**
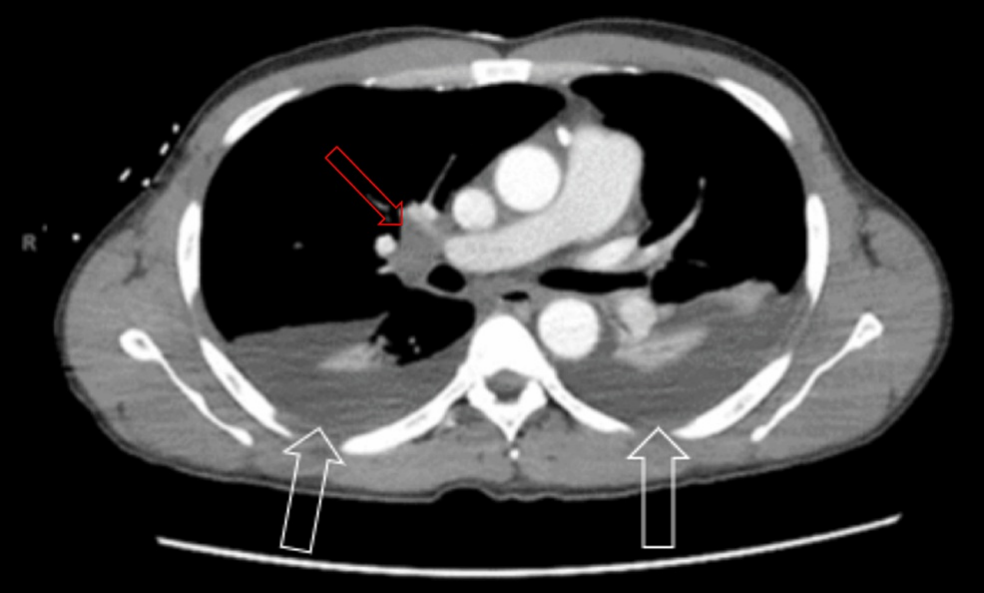
Bilateral pleural effusions (white arrows) with prominent right hilar lymph node (red arrow) visualized on CT

**Figure 3:**
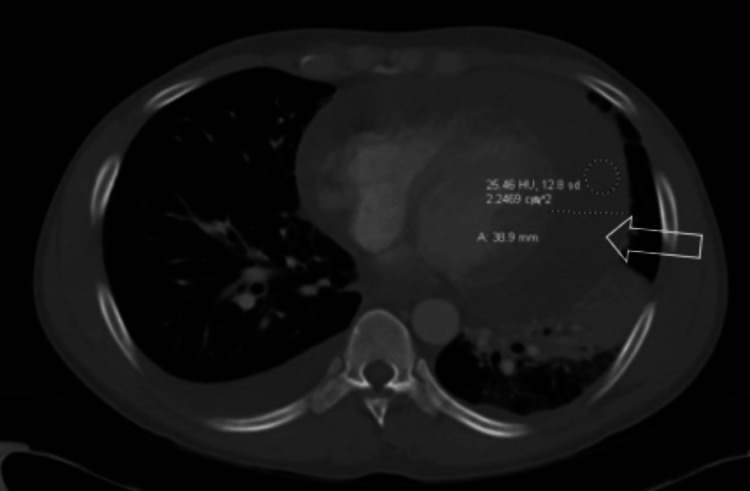
Large pericardial effusion visualized on CT

The initial ECG (Figure 1) demonstrated sinus tachycardia with a shortened PR interval and variation in the amplitude of the R wave consistent with electrical alternans. Computerized tomography (CT) with contrast of the chest, abdomen, and pelvis revealed large bilateral pleural effusions (Figure 2), a large pericardial effusion (Figure 3), and hepatic congestion with a nutmeg appearance. In addition, there was enhancing prominent mediastinal lymphadenopathy and an enlarged right hilar lymph node concerning for a neoplastic process.

**Figure 4:**
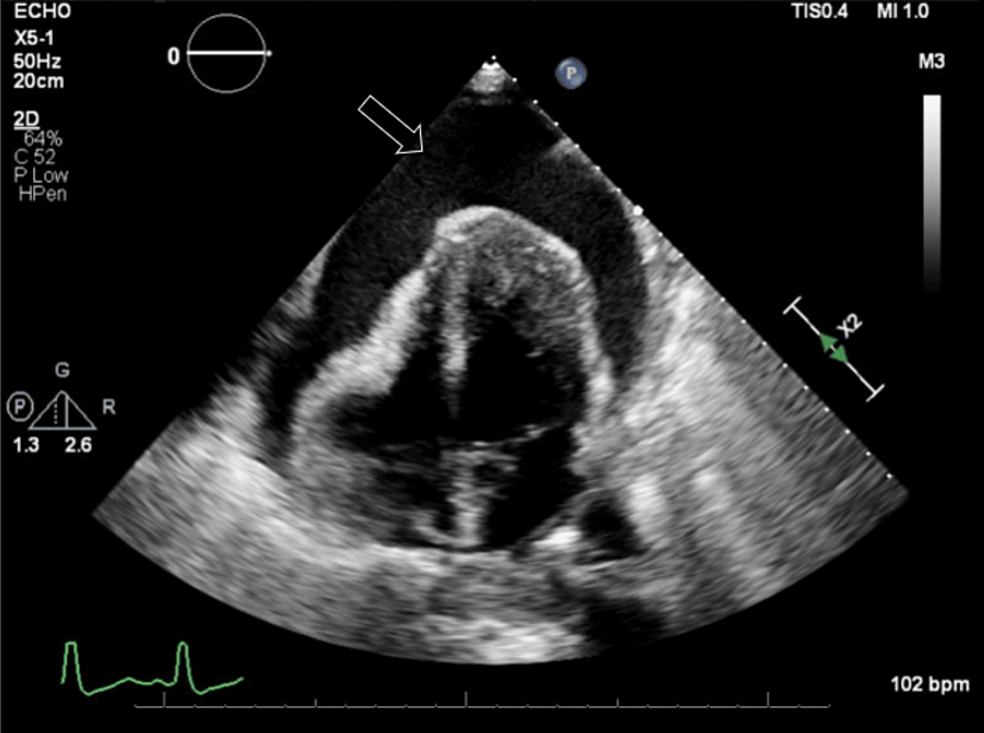
Large pericardial effusion visualized on echocardiography

**Figure 5:**
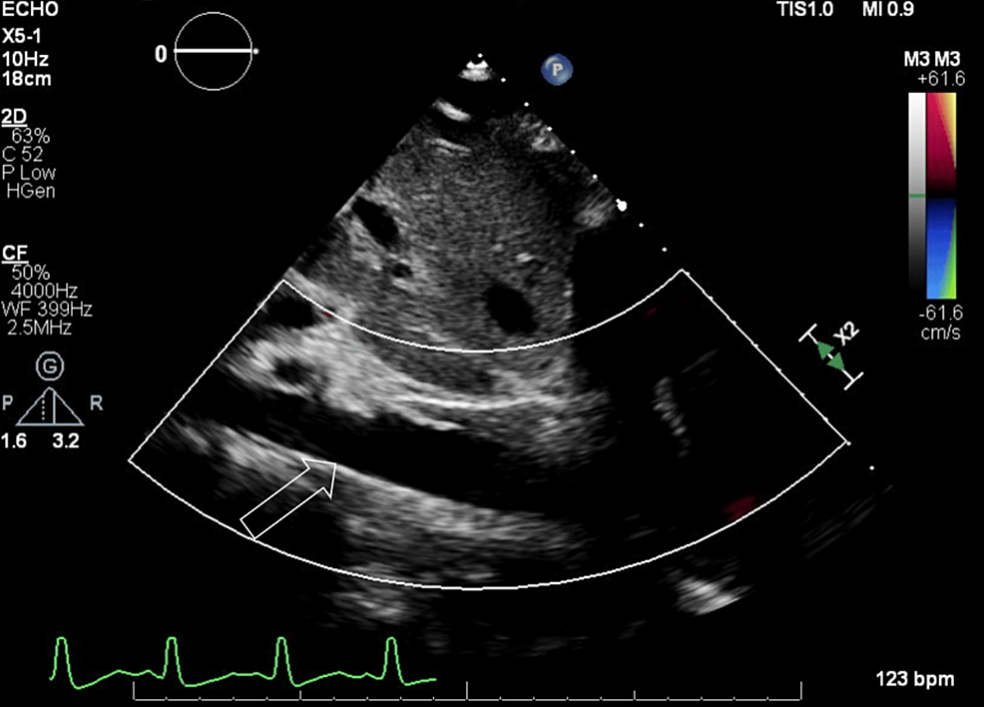
Dilated inferior vena cava visualized on echocardiography

An echocardiogram reported a left ventricular ejection fraction of 55-60%. There was a large pericardial effusion (Figure 4), and the inferior vena cava was dilated and lacked inspiratory variation (Figure 5), indicating cardiac tamponade. 

A pericardiocentesis was immediately performed. Approximately 1 liter of hemorrhagic fluid was removed. Cell counts of the pericardial aspirate can be found in Table 2. Malignant cells were found on cytological analysis of the fluid.

**Table 2:** Pericardial fluid analysis RBC: red blood cell, LDH: lactate dehydrogenase

Investigation	Patient values	Reference values
RBC	2,049,527/mm^3^	0
Nucleated cells	8,318/mm^3^	<200/mm^3^
LDH	5299 U/L	141-2613 U/L
Glucose	14 mg/dL	80-134 mg/dL

**Figure 6:**
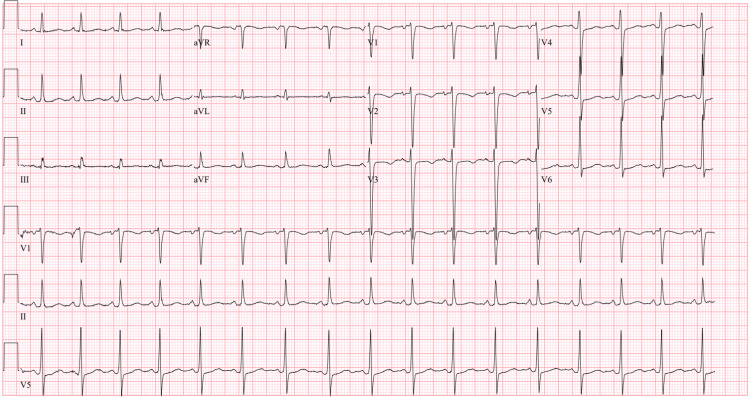
Repeat ECG obtained after pericardial fluid drainage without R wave amplitude variation

An ECG obtained following the procedure showed sinus tachycardia and resolution of the electrical alternans (Figure 6).

The patient was placed on colchicine and furosemide as adjuvant therapies.

**Figure 7:**
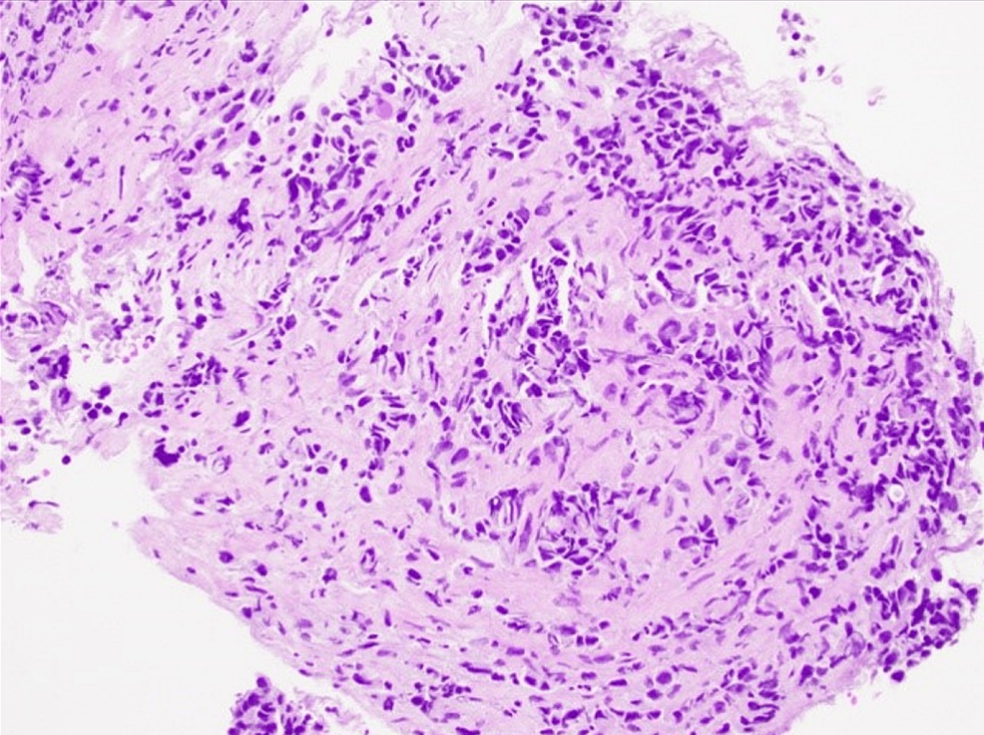
Hematoxylin and eosin-stained tissue section shows a poorly differentiated infiltrative non-small cell carcinoma (H&E; 200x)

**Figure 8:**
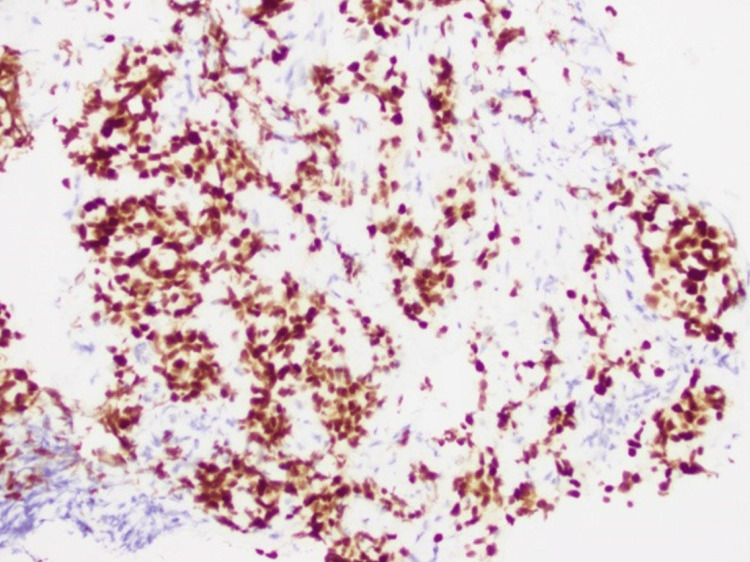
Keratin 7-positive immunohistochemistry staining favors pulmonary origin adenocarcinoma (IHC; 200x)

**Figure 9:**
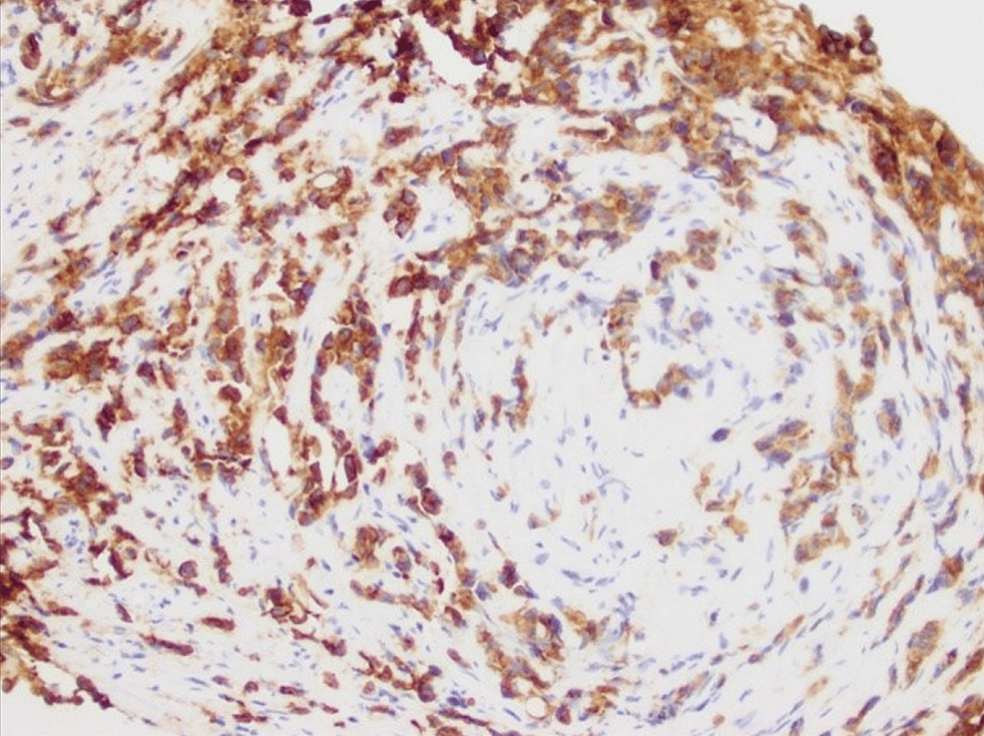
Thyroid transcription factor 1-positive immunohistochemistry staining favors pulmonary origin adenocarcinoma (IHC; 200x)

A CT of the chest with contrast two days after admission revealed prominent mediastinal lymphadenopathy and an enlarged, enhancing left supraclavicular lymph node. In addition, several small pulmonary nodules in the right lung were identified. The investigation continued with a biopsy of the right hilar lymph node. Pathological findings indicated poorly differentiated infiltrative non-small cell carcinoma (Figure 7) with immunohistochemistry staining, suggesting a pulmonary origin (Figures 8, 9).

Our patient’s symptoms progressively improved over the next few days. The aspartate aminotransferase (AST) and alanine aminotransferase (ALT) improved to 82 U/L and 265 U/L, respectively.

Oncology was consulted and made recommendations for treatment. The patient declined further intervention and decided to pursue outpatient palliative care. He was discharged from the hospital in stable condition.

## Discussion

Electrical alternans is an ECG finding often associated with large pericardial effusions [3]. The sign is defined as an alternating height of electrical complexes arising from the same pacemaking site. Any ECG wave can show this change, but it is most often observed with the QRS complex [1]. A mechanism that contributes to this finding is the pendular motion of the heart throughout the cardiac cycle from accumulated pericardial fluid [4]. Although electrical alternans is typically associated with pericardial effusion, it has also been observed in other pathologies, such as immediately preceding ventricular fibrillation and during catecholaminergic states [5]. If the finding is associated with pericardial effusion, it can disappear following adequate drainage [6].

Large pericardial effusions result in cardiac tamponade in about 25% of cases [7]. A weakened apical pulse, distant heart sounds, pulsus paradoxus, and narrow pulse pressure mark the syndrome. Cardiac tamponade results in a life-threatening state of reduced venous return and impaired diastolic functioning. The ensuing shock can be fatal [7].

There are many etiologies of pericardial effusion. Pericarditis, autoimmune disease, and malignancy are frequent causes. Malignant involvement of the pericardium is common, but the treatment of the malignancy itself is another possible culprit [8].

The diagnosis of cardiac tamponade requires a high degree of clinical suspicion. ECG can be a vital tool in diagnosis. Signs indicating cardiac tamponade include low QRS voltage, PR segment depression, sinus tachycardia, and electrical alternans. An echocardiogram could report a swinging heart, right atrial systolic collapse, right ventricular free wall diastolic collapse, enlarged and non-pulsatile inferior vena cava, and the presence of fluid in the pericardial space. Advanced imaging techniques are occasionally used. A CT or MRI may show deformed cardiac chambers and distention of the vena cava [9].

Cardiac tamponade requires immediate drainage to avoid life-threatening complications. Ultrasound-guided pericardiocentesis is an effective procedure to alleviate this condition. Fluid removal should be less than 1 liter to avoid right ventricular dilation that can result in profound hypotension. Other possible treatment options include subxiphoid surgical drainage, pericardial window technique, and left anterior minithoracotomy [10]. There is evidence that pericardiocentesis followed by continuous negative pressure drainage via a pigtail catheter can result in better patient outcomes [11].

The optimal management of malignant pericardial effusion is debated. The creation of a pericardial window is frequently used to prevent effusion recurrence. Despite this definitive procedure, the prognosis is extremely poor in the postoperative period. Most patients with malignant effusion experience only three months of survival [12]. Adjuvant therapy to prevent effusion recurrence includes systemic or intrapericardial antineoplastics or anti-inflammatory medications. Colchicine was used in our patient. This drug is highly beneficial in inflammatory pericardial effusion but has shown limited success in malignant effusion [13].

## Conclusions

Electrical alternans is a unique ECG finding often associated with large pericardial effusions. Our patient presented in cardiac tamponade with electrical alternans on ECG. After adequate pericardial fluid drainage, the sign resolved. Further evaluation resulted in a diagnosis of lung adenocarcinoma. Our patient decided to pursue palliative care in the outpatient setting after inpatient stabilization. Pericardial effusion resulting in cardiac tamponade has many etiologies. Although optimal chronic care of malignant pericardial effusion is debated, acute treatment requires drainage of pericardial fluid to avoid life-threatening complications.
